# Immunity to Distinct Sand Fly Salivary Proteins Primes the Anti-Leishmania Immune Response towards Protection or Exacerbation of Disease

**DOI:** 10.1371/journal.pntd.0000226

**Published:** 2008-04-16

**Authors:** Fabiano Oliveira, Phillip G. Lawyer, Shaden Kamhawi, Jesus G. Valenzuela

**Affiliations:** 1 Vector Molecular Biology Unit, Laboratory of Malaria and Vector Research, National Institute of Allergy and Infectious Diseases, National Institutes of Health, Rockville, Maryland, United States of America; 2 Centro de Pesquisas Gonçalo Moniz, FIOCRUZ, Salvador, Bahia, Brazil; 3 Intracellular Parasite Biology Section, Laboratory of Parasitic Diseases, National Institute of Allergy and Infectious Diseases, National Institutes of Health, Bethesda, Maryland, United States of America; Liverpool School of Tropical Medicine, United Kingdom

## Abstract

**Background:**

Leishmania parasites are transmitted in the presence of sand fly saliva. Together with the parasite, the sand fly injects biologically active salivary components that favorably change the environment at the feeding site. Exposure to bites or to salivary proteins results in immunity specific to these components. Mice immunized with *Phlebotomus papatasi* salivary gland homogenate (SGH) or pre-exposed to uninfected bites were protected against *Leishmania major* infection delivered by needle inoculation with SGH or by infected sand fly bites. Immunization with individual salivary proteins of two sand fly species protected mice from *L. major* infection. Here, we analyze the immune response to distinct salivary proteins from *P. papatasi* that produced contrasting outcomes of *L. major* infection.

**Methodology/Principal Findings:**

DNA immunization with distinct DTH-inducing salivary proteins from *P. papatasi* modulates *L. major* infection. PpSP15-immunized mice (PpSP15-mice) show lasting protection while PpSP44-immunized mice (PpSP44-mice) aggravate the infection, suggesting that immunization with these distinct molecules alters the course of anti-Leishmania immunity. Two weeks post-infection, 31.5% of CD4^+^ T cells produced IFN-γ in PpSP15-mice compared to 7.1% in PpSP44-mice. Moreover, IL-4-producing cells were 3-fold higher in PpSP44-mice. At an earlier time point of two hours after challenge with SGH and *L. major*, the expression profile of PpSP15-mice showed over 3-fold higher IFN-γ and IL-12-Rβ2 and 20-fold lower IL-4 expression relative to PpSP44-mice, suggesting that salivary proteins differentially prime anti-Leishmania immunity. This immune response is inducible by sand fly bites where PpSP15-mice showed a 3-fold higher IFN-γ and a 5-fold lower IL-4 expression compared with PpSP44-mice.

**Conclusions/Significance:**

Immunization with two salivary proteins from *P. papatasi*, PpSP15 and PpSP44, produced distinct immune profiles that correlated with resistance or susceptibility to Leishmania infection. The demonstration for the first time that immunity to a defined salivary protein (PpSP44) results in disease enhancement stresses the importance of the proper selection of vector-based vaccine candidates.

## Author Summary

In vector-borne diseases, the role of vectors has been overlooked in the search for vaccines. Nonetheless, there is a body of evidence showing the importance of salivary proteins of vectors in pathogen transmission. Leishmaniasis is a neglected vector-borne disease transmitted by sand flies. Pre-exposure to sand fly saliva or immunization with a salivary protein protected mice against cutaneous leishmaniasis. Using DNA immunization we investigated the immune response induced by abundant proteins within the saliva of the sand fly *Phlebotomus papatasi*. We found that one salivary protein protected while another exacerbated *L. major* infection, suggesting that the type of immune response induced by specific salivary proteins can prime and direct anti-Leishmania immunity. This stresses the importance of the proper selection of vector-based vaccine candidates. This work validates the powerful protection that can be acquired through vaccination with the appropriate salivary molecule and more importantly, shows that this protective immune response is efficiently recalled by sand fly bites, the natural route of transmission.

## Introduction

In leishmaniasis, phlebotomine sand flies transmit Leishmania parasites to a mammalian host by depositing the parasite in the skin during probing and feeding. Together with the parasite, sand flies deposit a repertoire of salivary components that assist the sand fly in getting a blood meal [1]. Some of these salivary proteins are immunogenic in humans, canids and mice [2]–[5]. Repeated exposure to sand fly salivary gland homogenate (SGH) or sand fly bites have been shown to protect mice to subsequent challenge with *Leishmania major* and SGH [6] or *L. major* infected sand flies [7].

The protective effect of insect saliva is not exclusive to sand flies and leishmaniasis. Animals pre-exposed to tick bites were protected from Borrelia infection [8] and from the fatal outcome of tularemia [9]. Moreover, immunization with a single tick salivary protein protected mice from the fatal outcome of encephalitis virus [10]. Furthermore, pre-exposure to mosquito bites protected mice against *Plasmodium berghei* infection [11] and more recently, immunization with the saliva of an aquatic insect (*Naucoris* genus) protected animals against *Mycobacterium ulcerans* infection [12].

To date, only two sand fly salivary proteins, Maxadilan from *Lutzomyia longipalpis* and PpSP15 from *Phlebotomus papatasi,* have shown promise as protective molecules against leishmaniasis [13],[14]. It is proposed that immunity to maxadilan neutralizes exacerbation of *L. major* infection [13], while immunization with PpSP15 results in protection of wild-type and B-cell deficient mice indicating that cellular immunity to PpSP15 is sufficient for protection [14]. Moreover, the protection observed by immunization with PpSP15 was associated with a DTH response [14]. More recently, Oliveira et al. investigated the IgG isotypes produced by DNA immunization with plasmids encoding distinct DTH-inducing sand fly salivary proteins and showed that some molecules produce IgG2a antibodies indicative of a Th1 response while others surprisingly produced IgG1, a marker for Th2 response in mice [15].

In this work we identified two additional DTH-inducing salivary proteins in *P. papatasi*, PpSP42 and PpSP44. Mice immunized with either of these molecules were not protected against *L. major* infection. Moreover, PpSP44-immunized mice showed aggravated lesions. This allowed us to explore how immunity to specific salivary proteins could affect the outcome of *L. major* infection. We show for the first time that an early adaptive immune response specific to a salivary protein is able to prime the anti-Leishmania immune response leading to protection or exacerbation of *L. major* infection. More importantly, this adaptive response is efficiently elicited by sand fly bites, the natural route of transmission.

## Methods

### Sand fly rearing and exposure to animals

*P. papatasi* Israeli strain sand flies were reared at the Walter Reed Army Medical Research Institute and at the Laboratory of Malaria and Vector Research, NIAID, NIH, as described elsewhere [14]. Preparation of salivary gland homogenate (SGH) and pre-exposure of mice (Charles River Laboratories Inc) to uninfected sand flies was carried out according to Valenzuela et al. [14] and Kamhawi et al. [7]. Experiments were performed using 6 to 8 weeks old C57BL/6 mice under pathogen free conditions. All animal studies were approved by the Animal Care and Use Committee at The National Institute of Allergy and Infectious Diseases.

### Construction of *P. papatasi* salivary DNA plasmids and immunization of mice

Ten DNA plasmids encoding to *P. papatasi* salivary gland-secreted proteins were cloned into the VR2001-TOPO vector and purified as previously described [15]. Mice were immunized intradermally in the right ear three times at two weeks intervals with 5 µg of DNA plasmid in 10 µl sterile water or with the equivalent of 0.5 sand fly salivary gland pairs in 10 µl PBS [15].

### Intradermal challenge with SGH and Leishmania parasites

Two weeks after the last DNA immunization, animals were challenged intradermally in the left ear with *P. papatasi* SGH (0.5 salivary gland pair/10 µl) to test for DTH inducing salivary proteins. For infection, a mixture of 0.5 pairs SGH and 500 *L .major* metacyclics in 10 µl (SGH-LM) was used to mimic the natural route of transmission. *L. major* clone V1 (MHOM/IL/80/Friedlin) was cultured in 199 medium with 10% heat-inactivated fetal bovine serum (HyClone), 100 U/ml penicillin, 100 µg/ml streptomycin, 2 mM L-glutamine and 40 mM Hepes.

### Ear thickness and lesion size

The ear thickness was measured 48 hours following intradermal injection of *P. papatasi* SGH. Values are represented as Δ ear thickness (ear thickness of experimental groups subtracted from the mean ear thickness of naïve mice 48 hours after injection with 0.5 pair of SGH). For measurements of Leishmania lesions, the largest diameter was recorded on a weekly basis. Ear thickness and lesion diameter were measured using a Digimatic caliper (Mitutoyo Corp.).

### Parasite load

Total genomic DNA was extracted from mice ears using the DNeasy tissue kit following the manufacturer's protocol (Qiagen). A total of 100 ng was amplified by real time PCR (LightCycler 480, Roche Diagnostics) using primers JW11 and JW12 [16] and 18S primers as a housekeeping gene with the FastStart Sybr green I kit (Roche). The standard curve was generated using DNA from naïve ears spiked with 10-fold serial dilutions of *L. major* DNA. Expression levels were normalized to 18S DNA and corrected for the weight of the whole ear. Values represent the relative number of parasites per ear.

### Intracellular Cytokines

Cells were recovered from the ear dermis as described previously [6]. Cells (5×10^6^) were stimulated with or without 100 µg soluble Leishmania antigen (SLA) for 12 hours. The cells were then stimulated with 20 ng PMA and 500 ng ionomycin, in the presence of monensin (2 µM final concentration) for 4 hours. For surface markers, cells were washed, incubated for 15 min at 4°C with 2.4G2 mAb to block FcγR, and stained with APC-Cy7 αCD4 (RM4-5) and APC-TCRβ chain (H57-597) for 20 min at 4°C. The cells were fixed, permeabilized (Cytofix/Cytoperm Plus; BD Pharmingen) and stained with PE-Cy7 αIFN-γ (XMG 1.2) and PE αIL-4 (11B11).The data were collected using a FACSArray (BD Biosciences) and analyzed with FlowJo software (Tree Star). The lymphocytes were gated using size, granularity and surface markers.

### GEArray

Expression profile of cytokines, chemokines, and related inflammatory genes was generated using the mouse inflammatory cytokines and receptor Oligo GEArray (OMM-011; Superarray). This array contains 112 genes representing cytokines, receptors and housekeeping genes. Two hours after challenge, total RNA was isolated from the left ears using QIAshredder (Qiagen) and RNeasy Mini Kit (Qiagen) following the manufacturer's instructions. RNA (6 µg) from a pool of seven ears was amplified and labeled with biotin 16-UTP (Roche Diagnostics) using the SuperArray TrueLabeling-RT Enzyme kit (Superarray). The resulting biotinylated cRNA was hybridized overnight to the Oligo GEArray® membrane. After washing and blocking the array membranes, alkaline phosphatase-conjugated streptavidin was added to the membrane followed by CDP-Star substrate. A chemiluminescent signal was acquired using the Image Station 2000 MM (Kodak). The data was analyzed using the GEArray Expression Analysis Suite (Superarray). Analysis parameters were set to local background correction and normalized to a set of housekeeping genes included in each membrane. Results were expressed as the fold increase in the intensity of the captured signal over the levels in naïve ears challenged with SGH-LM. Only genes showing a four-fold or higher change in expression compared to the naïve group in at least two of three independent experiments were considered.

### GEArray Validation

The genes that showed a four-fold or higher change in expression over control using the GEArray were validated by Real time PCR. Five µg of total RNA from mice ears was used for the synthesis of cDNA (Superscript III, Invitrogen) following the manufacturer's instructions. The cDNA was amplified with the 480 Master SYBR Green I mix (Roche Diagnostics) and gene specific primer sets for IFN-γ, IL-4, IL-5, TNF-α and IL-12Rβ2 (Superarray) using the LightCycler 480 (Roche Diagnostics). A standard curve for each set of primers was generated as recommended by the manufacturer. The expression levels of the genes of interest were normalized to endogenous 18S RNA levels. The results are expressed in fold change over naive ears challenged with SGH-LM.

### Statistical analysis

Statistical evaluation of the means of experimental groups was done using one-way analysis of variance followed by the Tukey-Kramer post-test. Data from parasite numbers were log transformed before conducting statistical tests. Significance was determined as *p*<0.05. All statistical tests and graphs were done using Prism-GraphPad version 5 (GraphPad Software Inc.).

## Results

### Immunization with PpSP15, PpSP42 and PpSP44 salivary proteins induces a specific DTH response

**Figure 1:**
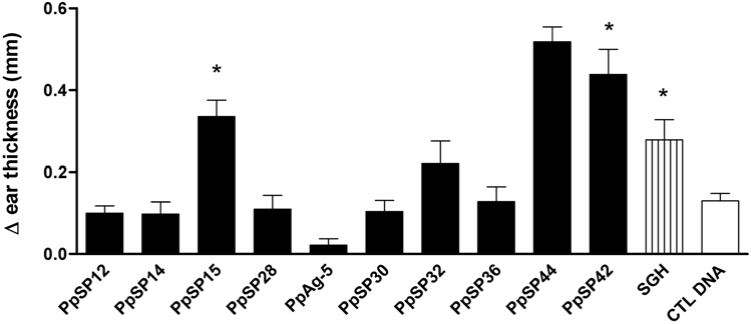
DNA immunization with PpSP15, PpSP42 and PpSP44 induces a DTH response after challenge with SGH. C57BL/6 mice were immunized three times at two week intervals with DNA plasmids coding for ten *Phlebotomus papatasi* salivary proteins, SGH and CTL DNA in the right ear. Two weeks later the left ear was challenged intradermally with 0.5 pairs of SGH. The DTH response was assessed using Δ ear thickness (ear thickness of experimental groups subtracted from the mean ear thickness of naïve mice) 48 hours after injection with 0.5 pairs of SGH. Bars represent the mean Δ ear thickness for 15 mice per group ± the SEM. Asterisks indicate statistical differences (*p*<0.05) compared to CTL DNA-immunized mice.

Of 10 different DNA plasmids coding for the most abundant *P. papatasi* salivary proteins [14],[17], PpSP12 (12-kDa protein; AF335485), PpSP14 (14-kDa protein; AF335486), PpSP15 (15-kDa protein; AF335487), PpSP28 (28-kDa protein; AF335488), PpAg5 (29-kDa protein; ABA54266), PpSP30 (30-kDa protein; AF335489), PpSP32 (32-kDa protein; AF335490), PpSP36 (36-kDa protein; AF261768), PpSP42 (42-kDa protein; AF335491), and PpSP44 (44-kDa protein; AF335492), only mice immunized with PpSP15, PpSP42 and PpSP44 DNA plasmids showed a statistically significant (*p*<0.05) DTH response 48 hours following challenge with SGH as measured by Δ ear thickness compared to control DNA-immunized mice (CTL DNA) (Fig. 1). However, immunization with PpSP12, PpSP14, PpAg5, PpSp32 and PpSP36 produced humoral responses (data not shown) indicating *in vivo* expression of the corresponding proteins. PpSP15 is a 15 kDa salivary protein of unknown function present only in sand flies [14],[17]. PpSP42 and PpSP44 are salivary proteins that belong to the Yellow family of proteins [17] with a predicted molecular weight of 42 and 44 kDa respectively.

### DNA immunization with distinct DTH-inducing salivary proteins can either promote or protect against *L. major* infection

**Figure 2:**
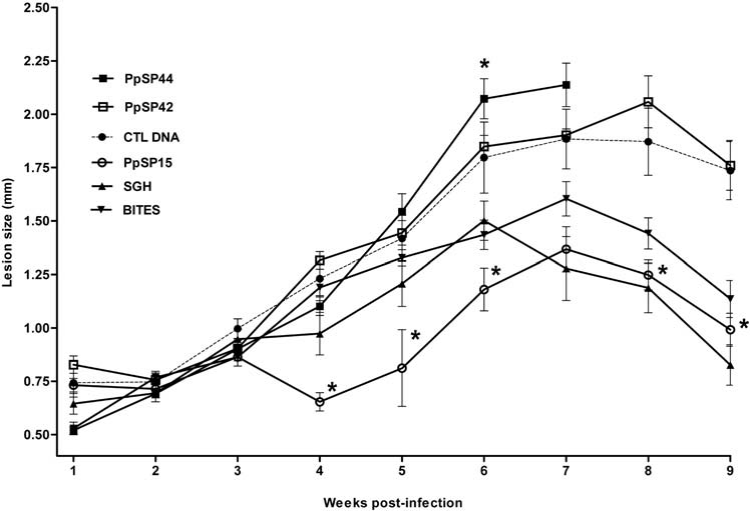
DNA immunization with distinct DTH-inducing salivary proteins modulates the course of infection with *L. major*. Mice immunized in the right ear with CTL DNA (•), PpSP15 (○), PpSP42 (□), PpSP44 (▪), SGH (▴) or pre-exposed to bites of uninfected sand flies (▾) were challenged in the left ear with 500 *L. major* metacyclics and 0.5 pairs of SGH. Due to the extensive ulceration of the ears in mice immunized with PpSP44, lesion size measurements could not be performed beyond seven weeks after challenge. The symbols represent the mean ± the SEM for ten mice per group. Asterisks indicate statistical significance (*p*<0.05) compared to mice immunized with CTL DNA. Data are representative of three different experiments.

Immunization with PpSP15 DNA or protein was previously shown to produce a DTH response and to protect animals from *L. major* infection [14]. Here we reaffirm the protective nature of PpSP15 but show that immunization with PpSP42 and PpSP44, the remaining DTH-inducing molecules, do not confer protection against *L. major* infection (Fig. 2). As predicted SGH or pre-exposure to uninfected sand fly bites also control *L. major* infection up to nine weeks post-challenge (Fig. 2). Mice immunized with PpSP44 exacerbated the infection showing progressive lesions that were predominantly ulcerative. The lesion size in this group was not measured beyond week seven due to extensive tissue damage (Fig. 2). This group was chosen for comparison to protected PpSP15-immunized mice for a better understanding of the contribution of anti-saliva immunity to the course of Leishmania infection.

### PpSP15-immunized mice show a three log reduction in parasite load compared to PpSP44-immunized mice following challenge with SGH-LM

**Figure 3:**
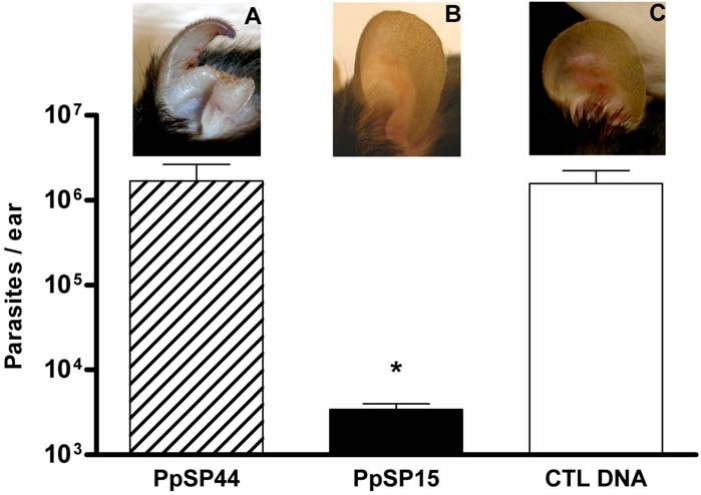
Protection or exacerbation of *L. major* infection in mice immunized with PpSP15 or PpSP44. Mice immunized with PpSP15, PpSP44 or CTL DNA were challenged with 500 *L. major* metacyclics and 0.5 pairs of SGH. The graph shows the number of parasites per ear at 11 weeks post-challenge as measured by Real time PCR. This result is representative of the parasite load at six and nine weeks post-challenge. Bars represent the mean ± the SEM for ten mice per group. Panels A–C reflect the pathology of the ears 11 weeks post-challenge in mice previously immunized with PpSP44 (A), PpSP15 (B) and CTL DNA (C). Asterisks indicate significance compared to mice immunized with CTL DNA (*p*<0.05). Data are representative of three independent experiments.

The parasite load was investigated at 2, 6, 9 and 11 weeks post-infection in PpSP15- and PpSP44-immunized mice. By 6 weeks post-infection, a significant decrease in parasite load was observed in mice immunized with PpSP15 compared with control DNA or PpSP44-immunized mice (data not shown). PpSP15-immunized mice maintained a 3 log reduction in parasite load up to 11 weeks post-infection. Panels A–C show representative ears of PpSP44-, PpSP15- and control DNA-immunized mice, respectively, 11 weeks post-infection (Fig. 3). Overall, the ears of PpSP15-immunized mice (Panel B) showed little to no tissue damage while those of PpSP44-immunized mice showed severe tissue erosion (Panel A). The ears of mice immunized with control DNA (Panel C) were intermediate showing ulcerated lesions with moderate tissue damage. Interestingly, the parasite loads were comparable in mice immunized with PpSP44 and control DNA, suggesting that the number of parasites in the ear of PpSp44-immunized animals was not entirely responsible for the extensive damage observed in these animals.

### PpSP15-immunized mice produced four-fold higher IFN-γ and three-fold lower IL-4 compared to PpSP44-immunized mice two weeks following challenge with SGH-LM

**Figure 4:**
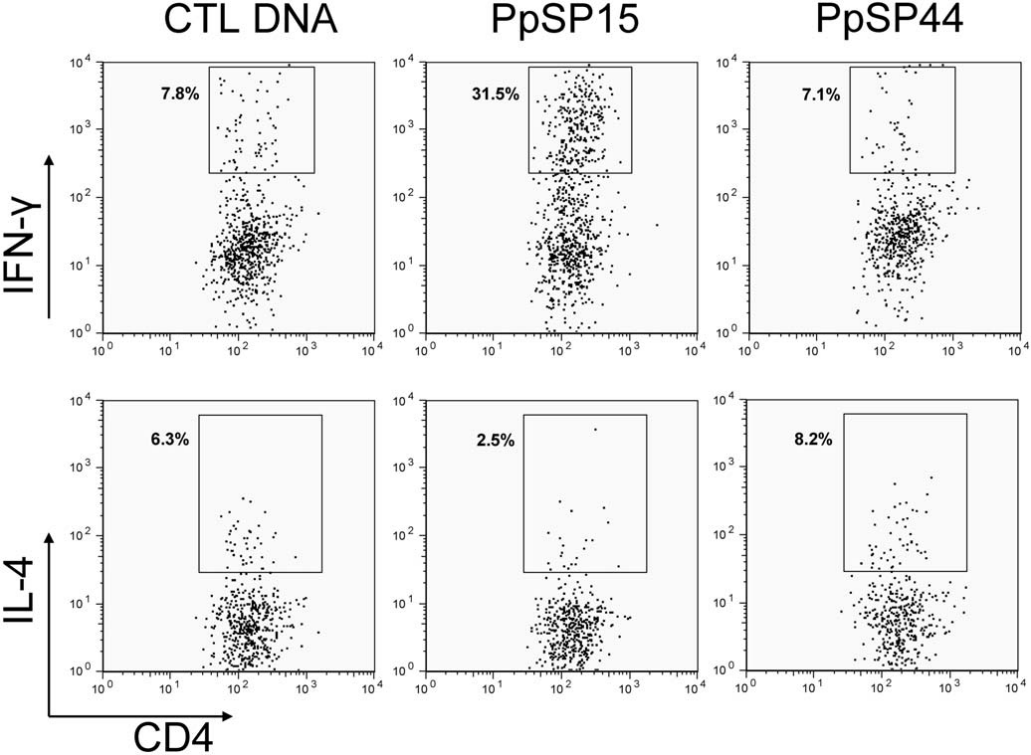
Frequency of CD4^+^-T cells producing IFN-γ or IL-4 in mice immunized with PpSP15 or PpSP44. Mice immunized in the right ear with CTL DNA, PpSP44 or PpSP15 were challenged in the left ear with 500 *L. major* metacyclics and 0.5 pairs of SGH. Two weeks after challenge, the percentage of CD4^+^ T cells producing IFN-γ or IL-4 were determined in cells recovered from the ear dermis (pools of three to five ears). Twelve hours after stimulation with SLA, cells were incubated for four hours with monensin, PMA and ionomycin and stained with CD4, TCRβ, IFN-γ and IL-4. The numbers represent the percentage of positive events. Data are representative of three independent experiments.

The observed protection and exacerbation of *L. major* infection in PpSP15- and PpSP44-immunized mice, respectively, correlates with the expression of IFN-γ and IL-4 by CD4^+^ T cells recovered from the ears of these mice two weeks after challenge with SGH-LM (Fig. 4). Following in vitro stimulation with soluble Leishmania antigen (SLA), 31.5% of CD4^+^ T cells in PpSP15-immunized mice produced IFN-γ compared to only 7.1% and 7.8% in mice immunized with PpSP44 and control DNA respectively (Fig. 4, top panels). IL-4 production was low in PpSP15-immunized mice (2.5% of CD4^+^ T cells). In comparison, 8.2% and 6.3% of CD4^+^ T cells produced IL-4 in mice immunized with PpSP44 and control DNA, respectively (Fig. 4, bottom panels). These data suggest that the immune response to distinct salivary proteins has a polarizing effect on the outcome of Leishmania infection.

### Differential induction of inflammatory transcripts in the ear of animals immunized with PpSP15 and PpSP44 two hours following challenge with SGH-LM

**Figure 5:**
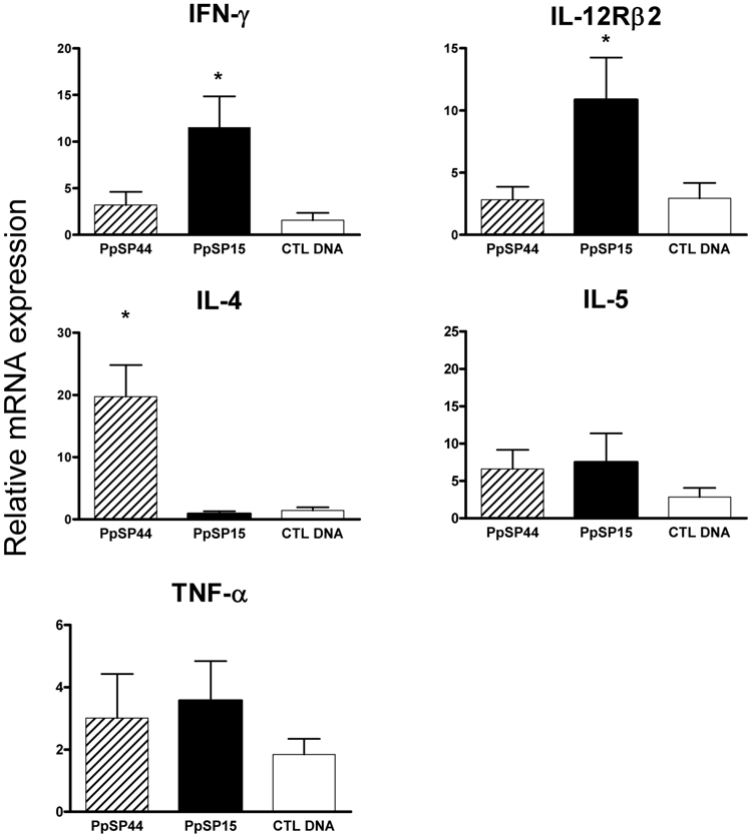
Early expression of cytokines after challenge with SGH-LM in CTL DNA, PpSP15- or PpSP44-immunized mice. Two hours after challenge with 500 *L. major* metacyclics and 0.5 pairs of SGH, expression of IFN-γ and IL-12Rβ2 was induced in mice immunized with PpSP15. In contrast, mice immunized with PpSP44 induced the expression of IL-4. Relative mRNA expression was determined by real time PCR and normalized to the18S housekeeping gene. Values represent the fold increase over naïve mice after challenge with SGH-LM. Bars represent the mean ± the SEM for 24 mice per group. Asterisks indicate statistical significance (*p*<0.05) between the PpSP15 and the PpSP44 experimental groups. Data represent the combined outcome of three independent experiments.

To understand the basis of the different outcomes of *L. major* infection in mice immunized with PpSP15 and PpSP44 we compared the early mRNA expression profiles of the inflammatory cytokines in the ears of these mice two hours following challenge with SGH-LM. Using the “Inflammatory Cytokines and Receptors” macroarray, transcripts showing a four-fold or higher change in signal intensity of gene expression compared to naïve controls were further analyzed and are presented in Table 1. PpSp15-immunized mice consistently produced high levels of IFN-γ and IL-12-Rβ2 and low levels of IL-4 and IL-5 (Table 1). In contrast, PpSP44-immunized mice produced high levels of IL-4 and IL-5 and baseline levels of IFN-γ transcripts. TNF-α transcripts were present at relatively high levels in mice immunized with PpSP15 and PpSP44 (Table 1). Real-time PCR was used to validate the results of the macroarray and showed that PpSP15-immunized animals induced a three-fold increase in IFN-γ and IL-12-Rβ2 messages compared to mice immunized with PpSP44 (*p*<0.05) (Fig. 5). Conversely, mice immunized with PpSP44 showed a 20-fold increase in the expression of IL-4 (*p*<0.005) and no significant expression of IFN-γ and IL-12-Rβ2 (Fig. 5). No significant difference was observed in the expression of IL-5 or TNF-α.

**Table 1:** Fold change in signal intensity of gene expression of inflammatory transcripts two hours following challenge with SGH-LM.

Transcripts	PpSP15			PpSP44		
	Exp1	Exp2	Exp3	Exp1	Exp2	Exp3
IFN-γ	5.73	10.5	2.55	1.02	1.00	1.36
IL-12Rβ2	6.26	1.72	4.02	1.17	1.00	1.00
TNF	8.71	1.68	11.95	6.91	3.72	1.42
IL-4	0.33	0.33	0.17	4.84	1.15	9.14
IL-5	0.64	0.53	0.19	2.9	5.2	3.49

### Mice immunized with PpSP15 and PpSP44 differentially induce IFN-γ and IL-4 in response to uninfected sand fly bites

**Figure 6:**
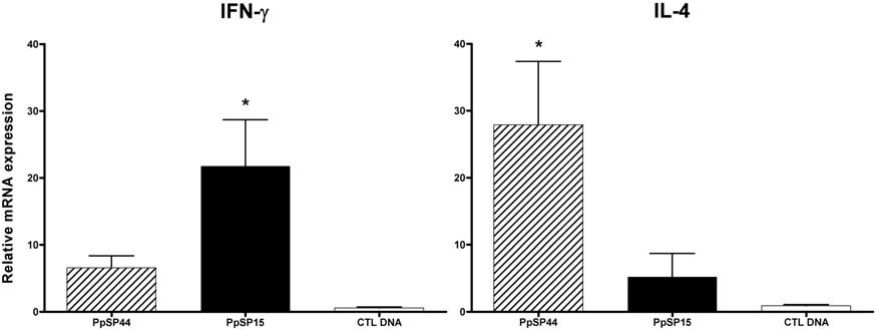
Sand fly bites efficiently recall the immune response in mice immunized with PpSP15 or PpSP44. Two hours after challenge with sand fly bites, expression of IFN-γ and IL-4 was compared in mice immunized with CTL DNA, PpSP15 or PpSP44. Relative mRNA expression was determined by real time PCR and normalized to the18S housekeeping gene. Values represent the fold increase over naïve mice after sand fly bites. Bars represent the mean ± the SEM for 16 mice per group. Asterisks indicate significance (*p*<0.05).

The amount of each salivary protein injected by sand flies during feeding is unknown. Therefore, we investigated whether the early induction of IFN-γ and IL-4 in mice immunized with PpSP15 and PpSP44, observed by challenge with SGH-LM, is reproducible by challenge with sand fly bites. In addition, uninfected sand flies were used to demonstrate that this response remains unchanged in the absence of parasites. Two hours following uninfected sand fly bites, mice immunized with PpSP15 showed a three-fold higher expression of IFN- γ and a five-fold lower expression of IL-4 compared with PpSP44-immunized mice (Fig. 6). There were no significant differences in the expression of IL-12Rβ2 or IL-5 amongst mice immunized with PpSP15, PpSP44 and control DNA (data not shown). This response shows that an adaptive immune response specific to distinct salivary proteins is inducible as early as two hours following sand fly bites and that the amount of salivary protein injected by the bite of a sand fly is sufficient to produce a specific and strong recall response in immunized animals.

## Discussion

It is established that a Th1 immune response and the production of IFN-γ are correlated with protection from *L. major* infection in C57BL/6 mice [18]. Conversely, a Th2 immune response is associated with susceptibility [18]. Earlier studies have demonstrated the potential of immunity to sand fly saliva in the control of Leishmania infection [6],[7],[13],[14]. More information is needed to define the immune profile induced by distinct salivary proteins and its specific effect on the outcome of disease. In this work, we demonstrate that DTH-inducing *P. papatasi* sand fly salivary molecules are not universally protective against *L. major* infection and that immunity to some can result in its exacerbation. Mice immunized with PpSP15 controlled the infection and had significantly lower parasite load compared to naïve mice, as previously reported [14]. In contrast, mice immunized with PpSP44 exacerbated the infection showing lesions with severe tissue erosion and maintaining a high number of parasites up to 11 weeks post-infection. This is the first account in which an immune response to a defined sand fly salivary protein results in disease exacerbation. Protection in PpSP15-immunized mice and exacerbation in PpSP44-immunized mice were correlated with an anti-Leishmania Th1 and Th2 immune response, respectively (Fig. 4). The anti-Leishmania immune response was characterized by a considerable increase in IFN-γ producing CD4^+^ T cells in PpSP15-immunized mice (over four-fold higher compared to control DNA- and PpSP44-immunized mice) and over three-fold lower IL-4 producing CD4^+^ T cells compared to PpSP44-immunized mice (Fig. 4). At this time point a small increase in the percent of CD4^+^ T cells producing IL-4 in PpSP44-immunized mice was detected compared to controls. Nevertheless, there is clear exacerbation both in lesion size and tissue pathology in PpSP44-immunized mice (Fig. 2, 3). We propose that the polarization of anti-Leishmania immunity towards a Th1 or Th2 response in these mice is the result of their prior immunization with DNA encoding the respective salivary proteins. Earlier studies have hypothesized that anti-saliva immunity leads to protection from *L. major* by the creation of a hostile environment that kills the parasite, acceleration and priming of the anti-Leishmania immunity, or a combination of both [7],[14]. Indeed, mice protected from *L. major* infection through pre-exposure to sand fly bites showed an increase in the frequency of ear epidermal cells producing IFN-γ and IL-12 six hours after challenge [7]. This rapid production of IFN-γ prompted us to investigate the expression profile of pro-inflammatory cytokines induced by PpSP15 and PpSP44 at an early time point (two hours) following challenge with SGH-LM. Macroarray results validated by real-time PCR showed that mice immunized with PpSP15 selectively induced transcripts associated with a Th1 immune response (IFN-γ and IL-12rβ2) and downregulated Th2 associated transcripts (IL-4). IL-12rβ2 is expressed on both activated Th1 CD4^+^ cells and NK cells [19]–[21]. Recently, it has been shown that NK cells could play a role in adaptive immunity [22] and may be the source of the early IFN-γ expression seen in PpSP15-immunized mice. Alternately, we cannot exclude the possibility that the up-regulation of IFN-γ expression is by specific CD4 memory T cells that are rapidly recruited to the site of infection. The cells that are responsible for the expression of IFN-γ at this early time point is currently under investigation. PpSP44- immunized mice that exacerbated *L. major* infection selectively induced IL-4 (a marker of Th2 differentiation) and did not upregulate IFN-γ showing the specificity of the observed immune responses to each of the salivary proteins. It should be noted that neither IFN-γ nor IL-4 were induced in the CTL DNA-immunized mice. Enhancement of Leishmania infection in mice pre-exposed to sand fly saliva was recently demonstrated for *Lu. intermedia* and *L. braziliensis* in a BALB/c model of infection [23]. Mice immunized with SGH of *Lu. intermedia* showed a low IFN-γ to IL-4 ratio that correlated with an enhanced disease profile [23]. It is possible that the immunodominant protein in the salivary repertoire of *Lu. intermedia* induces an immune response similar to that of PpSP44 resulting in the exacerbation of *L. braziliensis* infection in BALB/c mice. This is in contrast to the protection from *L. major* infection observed in BALB/c mice pre-exposed to *P. papatasi* bites or SGH [6],[7]. Interestingly, the molecular weight of a strongly antigenic salivary protein of *Lu. intermedia* is 45 kDa [23] corresponding to the molecular weight of PpSP44. This raises the question whether immuno-dominance of salivary proteins vary in different sand fly species. Saliva is composed of a repertoire of proteins and their overall effect is likely influenced by the sand fly species, the Leishmania species and the mammalian host resulting in an overriding exacerbative or protective immune profile. Stimulatory and suppressive immune responses to salivary molecules have been previously described in ticks [24]. Lymphocytes from tick resistant donors proliferated in response to tick salivary gland antigens demonstrating antigen-specific stimulation. However, their non-specific PHA-induced proliferation was significantly suppressed [24].

Since Leishmania is transmitted by sand fly bites we wanted to verify if the small amount of PpSP15 or PpSP44 injected by sand flies during feeding is able to recall the same level and type of immunity observed in response to challenge with SGH-LM. Moreover, we used uninfected sand flies to investigate whether this response is specific to the salivary molecules and is not influenced by the presence of Leishmania parasites in SGH-LM. Sand fly bites induced an early up-regulation of IFN-γ in PpSP15-immunized mice suggesting that this salivary protein can recall a protective Th1 response by the natural route of exposure. PpSP44-immunized mice also reproduced the response observed following challenge with SGH-LM and maintained a high expression of IL-4 and a low expression of IFN-γ (Fig. 6). Despite the fact that the above responses were elicited by uninfected sand fly bites, infected flies are expected to inject more saliva as a result of difficulty in feeding and increased probing activity [25]–[27]. This further confirms that an immune response specific to a salivary antigen that generates a DTH response with a Th1 profile is able to confer protection against *L. major* infection, independent of other confounding factors present in the complex feeding behavior of the sand fly. Recently, Vinhas et al. [28] demonstrated that PBMCs from normal volunteers pre-exposed to the bites of uninfected *Lu. longipalpis* produced IFN-γ following stimulation with SGH. IFN-γ production was also correlated with killing of *L. chagasi* parasites in a macrophage-lymphocyte autologous culture [28]. This demonstrates for the first time that humans can mount an anti-saliva cellular immune response that correlates with protection from Leishmania infection and emphasizes the need to identify the molecules in saliva that are responsible for this effect. Currently, there is no evidence that humans will mount a cellular immune response to either PpSP44 or PpSP15 proteins. Further studies are needed to elucidate the role of these salivary proteins in endemic areas.

Overall, these data suggest that the early induction of a distinct Th1-type immune response by salivary proteins is important for priming a protective immune response against Leishmania infection. Moreover, a DTH response to saliva or a salivary antigen by itself cannot be considered as a correlate of protection against Leishmania infection.

In conclusion, this paper clearly demonstrates that immunization with a particular salivary protein can have a profound modulatory effect on Leishmania infection. We believe that this immunization acts through the differential priming of anti-Leishmania immunity resulting in protection or susceptibility to disease.
